# The Effect of a Digital Mental Health Program on Anxiety and Depression Symptoms: Retrospective Analysis of Clinical Severity

**DOI:** 10.2196/36596

**Published:** 2023-10-03

**Authors:** Eldin Dzubur, Jessica Yu, Julia Hoffman, Stefanie Painter, Roberta James, Bimal Shah

**Affiliations:** 1 Teladoc Health Purchase, NY United States; 2 Duke University Medical School Durham, NC United States

**Keywords:** digital health, mental health, anxiety, depression, digital mental health, program usage

## Abstract

**Background:**

Evidence-based digital health programs have shown efficacy in being primary tools to improve emotional and mental health, as well as offering supplementary support to individuals undergoing psychotherapy for anxiety, depression, and other mental health disorders. However, information is lacking about the dose response to digital mental health interventions.

**Objective:**

The objective of the study was to examine the effect of time in program and program usage on symptom change among individuals enrolled in a real-world comprehensive digital mental health program (myStrength) who are experiencing severe anxiety or depression.

**Methods:**

Eligible participants (N=18,626) were adults aged 18 years and older who were enrolled in myStrength for at least four weeks as part of their employee wellness benefit program, who completed baseline, the 2-week, 2-month, and 6-month surveys querying symptoms of anxiety (Generalized Anxiety Disorder–7 [GAD-7]) and depression (Patient Health Questionnaire–9 [PHQ-9]). Linear growth curve models were used to analyze the effect of average weekly program usage on subsequent GAD-7 and PHQ-9 scores for participants with scores indicating severe anxiety (GAD-7≥15) or depression (PHQ-9≥15). All models were adjusted for baseline score and demographics.

**Results:**

Participants in the study (N=1519) were 77.4% female (1176/1519), had a mean age of 45 years (SD 14 years), and had an average enrollment time of 3 months. At baseline, participants reported an average of 9.39 (SD 6.04) on the GAD-7 and 11.0 (SD 6.6) on the PHQ-9. Those who reported 6-month results had an average of 8.18 (SD 6.15) on the GAD-7 and 9.18 (SD 6.79) on the PHQ-9. Participants with severe scores (n=506) experienced a significant improvement of 2.97 (SE 0.35) and 3.97 (SE 0.46) at each time point for anxiety and depression, respectively (*t*=–8.53 and *t*=–8.69, respectively; *P*s<.001). Those with severe baseline scores also saw a reduction of 0.27 (SE 0.08) and 0.25 (SE 0.09) points in anxiety and depression, respectively, for each additional program activity per week (*t*=–3.47 and *t*=–2.66, respectively; *P*s<.05).

**Conclusions:**

For participants with severe baseline scores, the study found a clinically significant reduction of approximately 9 points for anxiety and 12 points for depression after 6 months of enrollment, suggesting that interventions targeting mental health must maintain active, ongoing engagement when symptoms are present and be available as a continuous resource to maximize clinical impact, specifically in those experiencing severe anxiety or depression. Moreover, a dosing effect was shown, indicating improvement in outcomes among participants who engaged with the program every other day for both anxiety and depression. This suggests that digital mental health programs that provide both interesting and evidence-based activities could be more successful in further improving mental health outcomes.

## Introduction

Nearly 20% of adults in the United States report experiencing a mental illness [1]. Anxiety disorders are the most common mental health condition, affecting 18.1% of the adult population, while depression affects 6.7% of American adults and is the leading cause of disability in the country [2,3]. Nearly half of those diagnosed with depression also have a diagnosis of anxiety disorder, and while both are highly treatable, over 60% of those with anxiety and 35% of those with depression report not receiving treatment [3,4]. Regardless of an individual’s additional chronic or mental health conditions, evidence shows that anxiety also increases mortality by 43% and depression increases mortality by 50% compared to the general population [5,6]. Common barriers to receiving mental health treatment are stigma, access to mental health care, and affordability [4].

Digital mental health programs providing evidence-based self-help content, coaching, and other nontraditional forms of support are cost-effective, can reduce stigma-related reluctance to seek treatment, and have shown efficacy as both a primary and supplementary tool to improve emotional and mental health [7-11]. Digital mental health programs have been shown to produce significant reductions across mild, moderate, and severe anxiety and depression symptom severity groups with severe symptom participants showing the largest improvements [12]. Current literature has thoroughly explored digital mental health program outcomes and engagement through the number of logins, clicks, and time spent within the technology; however, information is lacking on dose response for digital mental health, which is defined as the amount of exposure that results in changed behavior [7,13-15].

While research has shown that those who use digital mental health features more frequently have greater positive outcomes, higher use does not universally equal greater results [16-19]. Purpose or motivation behind an individual using a digital mental health program may be more impactful on outcomes as program engagement can be influenced by the users’ attention, interest, and affect, which have been associated with acuity or clinical severity of symptoms [4,13,15,20]. Clinical severity can form a barrier to engagement with digital mental health platforms, specifically in individuals who experience depression [4]. The Generalized Anxiety Disorder–7 (GAD-7) and Patient Health Questionnaire–9 (PHQ-9) are validated instruments that provide a brief assessment of anxiety and depression symptom severity, and both instrument scoring manuals suggest increased levels of intervention with increased clinical severity [21]. Given the existing literature on the impact of engagement and clinical severity on outcomes, the objective of the study was to examine the effect of time in program, program usage, and dose response on symptom change among individuals enrolled in a comprehensive digital mental health program experiencing clinically severe anxiety or depression [12,16-19].

## Methods

### Study Design

A retrospective analysis of real-world data was performed to assess population characteristics, program usage, self-selected condition upon enrollment, and reason for program selection among adults aged 18 years and older enrolled in myStrength, a digital mental health program for a wide variety of mental health concerns. Participants’ GAD-7 and PHQ-9 scores at baseline, 14 days, 60 days, and 180 days were analyzed to determine the effect of average weekly program usage on subsequent GAD-7 and PHQ-9 scores for participants with scores indicating severe anxiety (GAD-7≥15) or depression (PHQ-9≥20).

### Program Description

The myStrength program is a web- and mobile-based mental health platform, which includes over 1000 digital activities, which provides education and delivers evidence-based resources to support challenges including anxiety, depression, insomnia, chronic pain management, stress, and substance abuse disorder through interactive applications. It integrates empirically proven psychotherapy models, such as cognitive behavioral therapy, motivational interviewing, acceptance and commitment therapy, positive psychology, and mindfulness along with mood tracking, sharing of community and personal inspirations, and a searchable library of mental health, wellness, and well-being resources. The member’s experience is highly personalized, based on individual interests, and can be used as a stand-alone tool or in conjunction with in-person, professional mental health care. To foster engagement members are sent weekly mental health-focused emails encouraging them to log in and use the tools. Individual activities are meant to take no more than 5-10 minutes to complete.

### Program Enrollment

During registration, members provided basic demographic information, including birthdate and gender, and self-selected their reason for enrolling in myStrength. Reasons for enrolling include “just curious,” “could use a boost,” “struggle with some things,” and “mental illness.” After selecting their reason for enrolling, the member self-selected a clinical concern (eg, managing depression, pregnancy and early parenting, drug or alcohol recovery) as their focus area. Members could choose multiple focus areas. Based on the members’ selected focus areas and content preferences, different programs and activities were suggested within the platform. Following focus area selection, members reported their current emotional state using a Likert scale. Clinical assessments were then offered through the mobile app, including GAD-7 and PHQ-9, and the enrollment process was completed. Members were prompted to reassess their symptoms with clinical assessments at 14, 30, 60, 180, and 365 days, and push notifications were sent if enabled on the member’s phone.

### Participants

**Figure 1:**
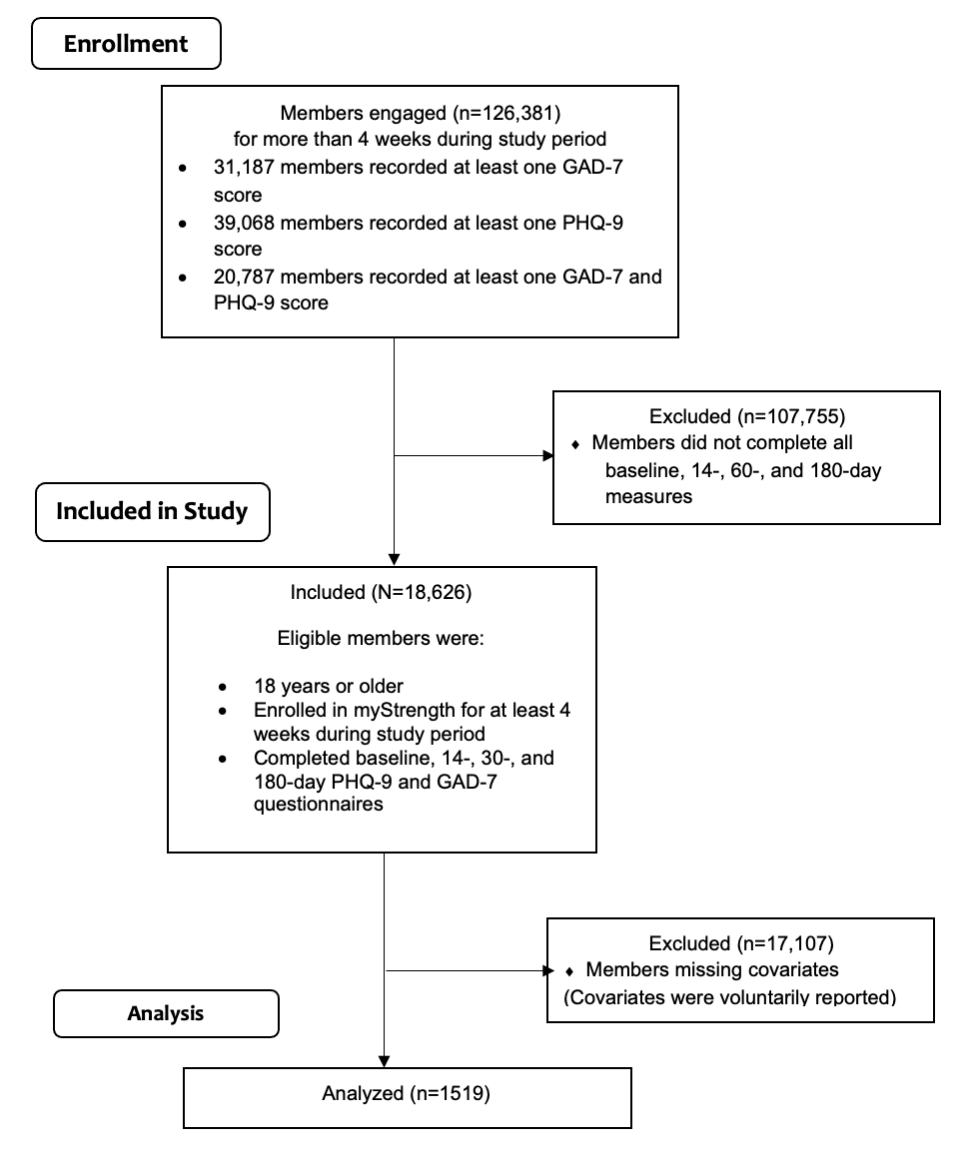
myStrength dosing study flow diagram. GAD-7: Generalized Anxiety Disorder–7; PHQ-9: Patient Health Questionnaire–9.

During the study period, 126,381 members were enrolled in myStrength for greater than 4 weeks, as part of their employee wellness benefit program and logged into the program at least once. Among these members, 31,187 had at least one GAD-7 score, 39,068 had at least one PHQ-9 score, and 20,787 had both GAD-7 and PHQ-9 scores. Eligible study participants included members (N=18,626) aged 18 years and older enrolled in myStrength for at least four weeks, who completed baseline, 14-day, 60-day, and 180-day surveys querying anxiety (GAD-7) and depression (PHQ-9). Participants were not excluded if they failed to complete all questionnaires, and no other a priori exclusion criteria were applied. The 6-month follow-up period started upon the member enrolling in the program. Members missing covariates were removed throughout analyses (N=1519). Figure 1 presents the study flow diagram.

### Ethics Approval

Institutional review board (IRB) approval was granted by Aspire IRB (#520160099), a third-party review board. All participants provided consent to participate during program enrollment, and guidelines were followed as outlined in the Declaration of Helsinki. All study data were stored in HIPAA (Health Insurance Portability and Accountability Act)-compliant, secure servers and were deidentified prior to analysis. Participants were not compensated for their participation in the study.

### Measures

#### Overview

GAD-7 and PHQ-9 were selected to measure symptoms and severity of anxiety and depression for this study as reliable and validated instruments extensively demonstrated within the scientific literature, known and commonly used tools within health care settings, and a brief design easily integrated into a digital health application. In addition to GAD-7, PHQ-9, and program activity (as defined below), age (years), gender (female vs not female), and days enrolled in the program were used as covariates in the model; participants without these covariates were excluded from analyses.

#### Generalized Anxiety Disorder–7

The GAD-7 is a scientifically validated, 7-item self-report anxiety questionnaire that assesses the degree to which a person has been feeling nervous, anxious, unable to stop or control worry, worrying too much about a variety of things, having trouble relaxing, restlessness, irritability, and afraid of something occurring [22]. Each item is scored as 0=not at all, 1=several days, 2=more than half the days, or 3=nearly every day, and then all items are totaled [23]. Mild anxiety has a score of 5 to 9, moderate anxiety has a score of 10 to 14, and severe anxiety has a score of ≥15; however, any score ≥10 is recommended for further evaluation [22].

#### Patient Health Questionnaire–9

The PHQ-9 is a scientifically validated, 9-item self-report questionnaire that assesses the degree to which a person has been experiencing symptoms of depression over the past 2 weeks. Major depression is diagnosed if ≥5 of the 9 depressive symptoms are selected as present for a minimum of 50% of the days within the past 2 weeks [24]. Each questionnaire item can be scored from 0 (not at all) to 3 (nearly every day) for defining severity [25]. PHQ-9 final scores are defined as “no depression” at 0-4, “mild depression” at 5-9, “moderate depression” at 10-14, “moderately severe depression” at 15-19, and “severe depression” at ≥20 [25].

#### Program Activity

Program activity usage is defined by the number of myStrength activities an individual engaged with per week, including both completed activities as well as activities that were not fully completed. The number of activities an individual engaged with was aggregated to the day level.

### Statistical Analysis

myStrength historical user data, including baseline demographics, program activity, and patient-reported surveys were retrieved from the Teladoc Health servers (myStrength is owned by Teladoc Health, Inc). Given lagging and data structure, a maximum of *t*–1 measurements were used per individual, where *t* represents the number of time periods with clinical assessments for up to 4 total time periods inclusive of baseline data. Furthermore, as a result of the unbalanced spacing in GAD-7 and PHQ-9 measurements, the program activity metric was normalized to the length of time between each measurement period (14 days, 46 days, and 120 days) and aggregated to the week level.

A generalized linear mixed model (GLMM) was used to test the main effect of program activity use and time on GAD-7 and PHQ-9 sum scores [26]. Age, gender, baseline GAD-7 or PHQ-9, the total number of days in the program (person level), and whether they endorsed anxiety or depression or both as a focus area were entered as covariates. Program activity use was disaggregated into between-subject (BS, deviation from the group mean) and within-subject (WS, deviation from person-level mean) variance components [26]. A subsequent GLMM was used to test the interaction of program activity use and time on GAD-7 and PHQ-9 sum scores, controlling for covariates. Last, individuals with severe scores on the GAD-7 and PHQ-9 were examined using the same set of main effects and interaction models for their respective subgroups.

## Results

### Member Characteristics and Program Usage

Participants were on average 45 years old and 77.4% female (1176/1519) with nearly 90 days on the myStrength program. Anxiety (1218/1519, 80%) and depression (1152/1519, 76%) were the most selected conditions at enrollment, with the most common reason for enrolling into the program being “I struggle sometimes” (694/1519, 46%). Over the study period, individuals completed an average of 2 measurements, with activities ranging from 1 to 3 activities per week depending on the measurement period. Participants in the study reported average baseline GAD-7 and PHQ-9 scores of 9.4 and 11.0, respectively. Table 1 provides further descriptive statistics and data on program usage.

**Table 1:** Member characteristics and program usage (N=1519).

Characteristic			Values
**Member characteristics**			
	Age (year), mean (SD)		45.25 (14.42)
	**Gender, n (%)**		
		Female	1176 (77.4)
		Male	343 (22.6)
**Program engagement** **, mean (SD)**			
	Days on program		88.12 (65.77)
	GAD-7^a^/PHQ-9^b^ measurements completed		2.34 (0.59)
	**Enrollment conditions, n (%)**		
		Anxiety	1218 (80.2)
		Depression	1152 (75.8)
		Meditation	220 (14.1)
		Stress reduction	1135 (74.7)
		Insomnia	937 (61.7)
		Chronic pain	416 (27.4)
	**Reason for program uptake, n (%)**		
		Challenge	381 (25)
		Curious	200 (13.2)
		Life is good	244 (16.1)
		Struggle	694 (45.7)
**Baseline measures^c^, mean (SD)**			
	GAD-7		9.39 (6.04)
	PHQ-9		11.0 (6.6)
**14-day measures^c^ (n=682), mean (SD)**			
	GAD-7		8.07 (5.76)
	PHQ-9		9.33 (6.45)
	Activities per week		2.89 (5.01)
**60-day measures^c^ (n=886), mean (SD)**			
	GAD-7		7.91 (6.17)
	PHQ-9		9.14 (6.58)
	Activities per week		1.18 (3.00)
**180-day measures^c^ (n=460), mean (SD)**			
	GAD-7		8.18 (6.15)
	PHQ-9		9.18 (6.79)
	Activities per week		0.88 (2.29)

^a^GAD-7: Generalized Anxiety Disorder–7.

^b^PHQ-9: Patient Health Questionnaire–9.

^c^The GAD-7 and PHQ-9 mean scores do not deviate from the median to imply substantial skewness at the study timepoints and present approximately a single-point deviation from the mean, providing valuable insight into the variance of the outcome.

### Program Engagement and GAD-7 and PHQ-9 Scores

As seen in Table 2, for each additional activity per week a member had engaged with greater than other members throughout the program (BS effect), there was a statistically significant reduction of 0.12 GAD-7 and 0.14 PHQ-9 scores at any timepoint (*P*s<.001). For each additional activity per week a member had engaged with greater than their own average (WS effect), there was a statistically significant reduction of 0.08 GAD-7 and 0.10 PHQ-9 scores at the subsequent timepoint (*P*s<.001). Members had a significant improvement of 1.05 and 1.38 points at each time point for anxiety and depression, respectively (*P*s<.001). Table 2 also indicates the interaction of time by program activity, significant at the BS level for both GAD-7 and PHQ-9 (*P*s<.05). Multimedia Appendix 1 presents the results of mixed effects models testing the effect of activity engagement on GAD-7 and PHQ-9 scores on the complete sample.

**Table 2:** Results of mixed effects models testing the effect of activity engagement on Generalized Anxiety Disorder–7 (GAD-7) and Patient Health Questionnaire–9 (PHQ-9) scores (N=1519).

Characteristic			Measure (outcome)														
			GAD-7								PHQ-9						
			B		SE	*t* value		*P* value		B			SE		*t* value		*P* value
Age (year)			–0.01		0.00	–2.91		<.001		–0.01			0.00		–1.76		.08
Gender (female)			0.24		0.15	1.57		.12		0.13			0.17		0.78		.43
Baseline measure			0.77		0.01	63.91		<.001		0.78			0.01		65.67		<.001
Timepoint			–1.05		0.13	–7.84		<.001		–1.38			0.15		–9.42		<.001
Days on program			0.01		0.00	4.45		<.001		0.01			0.00		4.79		<.001
Anxiety (yes)			0.56		0.18	3.21		<.001		0.50			0.18		2.72		.010
Depression (yes)			0.54		0.16	3.43		<.001		0.57			0.18		3.13		<.001
Activities per week (BS^a^)			–0.12		0.03	–3.73		<.001		–0.14			0.04		–3.97		<.001
Activities per week (WS^b^)			–0.08		0.03	–2.83		<.001		–0.10			0.03		–3.20		<.001
**Interactions**																	
	Timepoint × activities (BS)	–0.12		0.03		–3.45	<.001		–0.09			0.04		–2.51		.01	
	Timepoint × activities (WS)	0.00		0.04		0.04	.97		0.00			0.05		0.08		.93	

^a^BS: between-subject.

^b^WS: within-subject.

Table 3 displays the main effect and interaction models for the subgroup analysis for severe participants (GAD-7≥15 and PHQ-9≥15). Among members with severe GAD-7 scores (N=305), each additional activity per week of engagement compared to other members with severe GAD-7 scores (BS effect) was associated with a statistically significant reduction of 0.22 in GAD-7 score at any timepoint (*P*<.05). For those members with severe GAD-7 scores, each additional activity per week greater than one’s own average (WS effect) was associated with a statistically significant reduction of 0.27 in GAD-7 score at the subsequent timepoint (*P*<.001). Among members with severe PHQ-9 scores, each additional activity per week greater than one’s own average (WS effect) was associated with a statistically significant reduction of 0.25 in PHQ-9 score at the subsequent timepoint (*P*<.05). Members with severe scores had a significant improvement of 3.97 and 2.97 points at each time point for anxiety and depression, respectively (*P*s<.001). Table 3 also indicates the interaction of time by program activity, significant at the WS level for members with severe GAD-7 scores (*P*=.01). Multimedia Appendix 2 provides the results of mixed effects models testing the effect of activity engagement on GAD-7 and PHQ-9 scores for participants with severe scores on complete sample.

**Table 3:** Results of mixed effects models testing the effect of activity engagement on Generalized Anxiety Disorder–7 (GAD-7) and Patient Health Questionnaire–9 (PHQ-9) scores for participants with severe scores (GAD-7≥15 and PHQ-9>15).

Characteristic		Measure (outcome)								
		GAD-7 (n=305)					PHQ-9 (n=201)			
		B	SE	*t* value	*P* value	B		SE	*t* value	*P* value
Age		–0.02	0.01	–1.76	.08	–0.01		0.02	–0.58	.56
Gender (female)		0.54	0.45	1.20	.23	0.22		0.55	0.40	.69
Baseline measure		0.77	0.11	7.03	<.001	1.00		0.12	8.46	<.001
Timepoint		–2.97	0.35	–8.53	<.001	–3.97		0.46	–8.69	<.001
Days on program		0.03	0.01	4.25	<.001	0.03		0.01	3.63	<.001
Anxiety (yes)		0.78	0.94	0.83	.41	1.45		0.82	1.76	.08
Depression (yes)		0.09	0.60	0.14	.89	1.73		1.49	1.16	.25
Activities per week (BS^a^)		–0.22	0.09	–2.57	.01	–0.11		0.10	–1.04	.30
Activities per week (WS^b^)		–0.27	0.08	–3.47	<.001	–0.25		0.09	–2.66	.01
**Interactions**										
	Timepoint × activities (BS)	0.14	0.09	1.57	.12	0.12		0.11	1.11	.27
	Timepoint × activities (WS)	0.36	0.13	2.75	.01	0.01		0.14	0.08	.94

^a^BS: between-subject.

^b^WS: within-subject.

## Discussion

### Principal Findings

Our study highlights improved outcomes and dose-response engagement in a real-world digital mental health program. Over 6 months, results from the study found a trend toward clinically impactful change in anxiety and depression scores, suggesting that consistent engagement in digital mental health programs can maximize clinical impact. Moreover, additional engagement of 4 times per week (approximately every other day) in a program was associated with improvement in either anxiety or depression scores, implying that digital mental health programs that provide both interesting and evidence-based activities to further improve mental health outcomes are more likely to lead to clinical improvement. Last, a greater magnitude of improvement was seen within the clinically severe sample compared to the total population, which supports findings from previous literature that individuals with clinically severe anxiety and depression can see greater benefits when engaged in digital mental health programs.

The established benchmark for clinically meaningful change for both GAD-7 and PHQ-9 is a 5-point reduction in scores [27]. Study participants with severe scores had a 3.97 reduction in GAD-7 scores at each timepoint and a 2.97 reduction in PHQ-9 scores at each timepoint. Therefore, participants experienced a trend towards clinically meaningful change from baseline to 2 weeks, 2 weeks to 2 months, and 2 months to 6 months. Most notably, participants on average experienced a clinically meaningful change in GAD-7 and PHQ-9 scores by 2 months.

Previous literature has extensively provided a variety of engagement definitions and assessed adherence and related outcomes [13,15,20]. While higher engagement leading to more positive outcomes is nearly a unanimous finding among studies, nonadherence remains an unsolved issue for researchers [13]. Nonadherence, however, is defined by program developers versus user needs, and digital mental health programs are shown to be used when mental health symptoms are high instead of a predetermined schedule or consistent basis [13,18,20]. Due to the variable nature of mental health symptoms, some studies show that total usage does not always equate to better outcomes and quality of usage could be more impactful than quantity [16,20,28,29].

User experience and motivation for enrolling in a digital mental health program are affected by mental health symptoms and have been described as a key part of engagement [4,15]. Clinical severity can increase interest in engaging with a digital mental health program, but also has been shown to produce barriers when an individual becomes tired or does not feel a connection to the intervention [4]. Targeting the most beneficial and change-eliciting aspects of a digital mental health program is suggested as more important than overall usage to encourage change when a user’s interest is high [4,20]. Therefore, digital mental health programs should focus on assessing reasons for enrollment, symptom severity, and impact of program features. These 3 foci will help understand users’ need for engagement, desire to engage, use that need and desire to increase motivation, and collect data on the effectiveness of particular features to drive product enhancement. In addition, continual assessment of symptom severity and effectiveness of program features for feature recommendations and dose-response outcomes should be considered.

Our study approaches engagement from the user end to quantify a dose response to digital mental health program features on anxiety and depression outcomes that can be translated to other digital mental health programs. Understanding dose response for mental health conditions can help redefine consistent engagement and adherence based on the variable needs of the user over time, specifically in individuals with greater clinical severity at baseline. Defining consistent engagement within a digital mental health program could aid in setting user expectations, driving engagement, and supporting progress monitoring. While subjective engagement remains a key factor, it is imperative for programs to understand dose response to develop scalable personalized engagement features to encourage program usage when users are experiencing higher levels of symptoms. Personalized recommendations for engagement could include, but are not limited to, reminders to log into a program a specific number of times per week or to complete a specific number of activities per week depending on a person’s severity of symptoms, gamification, providing engagement rewards, and developing campaigns and challenges to motivate usage.

### Strengths and Limitations

Strengths of the study include repeated assessment of anxiety and depression over the course of 6 months with real-time measurement of intervention usage in a real-world population, and robust statistical modeling to determine both within- and between-person effects. The study was limited primarily by its retrospective observational design; therefore, individuals who were included in the study were likely more engaged and more likely to respond to mental health questionnaires. Moreover, those who did not engage with the program did not respond to the questionnaires and were thus not included in the study. While the GLMM is robust to unbalanced data sets and missing data in general, there are still gaps in understanding the effects of activity engagement (or lack thereof) on individuals who did not complete GAD-7 or PHQ-9. Hence, the study represents findings from individuals who likely benefited most from the intervention, given the implied nonengagement of those not responding to mental health questionnaires. Utilizing more frequent measurement periods, such as ecological momentary assessment, may reduce the impact of this limitation on future studies. Similarly, future work on the frequency of time spent on activities, click-throughs, and other meta-data that were not available during this study period can be examined to gain additional insight into participant engagement. Furthermore, the study was limited by sample size.

### Conclusions

Clinically significant reductions in anxiety and depression were present at 6 months in members enrolled in a digital mental health program. As we refine our understanding of the optimal dose response in digital mental health for conditions such as anxiety and depression, we must also refine our approach for motivating individuals to explore and use evidence-based products. Future research should focus on the user experience, and most specifically, the types of features and enhancements that draw people to use the tools provided and the types of incentives and rewards that encourage people to remain engaged. Additionally, while examining different activity types was not within the scope of this study, future studies could involve stratifying digital activities into different content types and determining how different activity types affect clinical outcomes. Such research would be another step towards increasing access to and engagement in novel digital mental health solutions.

## Appendix

Multimedia Appendix 1 Results of mixed effects models testing the effect of activity engagement on Generalized Anxiety Disorder–7 (GAD-7) and Patient Health Questionnaire–9 (PHQ-9) scores.

Multimedia Appendix 2 Results of mixed effects models testing the effect of activity engagement on Generalized Anxiety Disorder–7 (GAD-7) and Patient Health Questionnaire–9 (PHQ-9) scores for participants with severe scores.

## Abbreviations

BS: between-subject
GAD-7: Generalized Anxiety Disorder–7
GLMM: generalized linear mixed models
HIPAA: Health Insurance Portability and Accountability Act
IRB: institutional review board
PHQ-9: Patient Health Questionnaire–9
WS: within-subject
