# Supplementary material for: The Effect of a Digital Mental Health Program on Anxiety and Depression Symptoms: Retrospective Analysis of Clinical Severity
Source: JMIR Form Res. 2023 Oct 3;7:e36596. doi: 10.2196/36596 (PMC10582814; doi:10.2196/36596)
Supplement: Multimedia Appendix 1 [file formative_v7i1e36596_app1.docx]

Results of mixed effects models testing the effect of activity engagement on GAD-7^a^ and PHQ-9^b^ scores on full sample (N=18,626).

| Characteristic | | Measure (Outcome) | | | | | | | |
| --- | --- | --- | --- | --- | --- | --- | --- | --- | --- |
|  |  | GAD-7^a^ | | | | PHQ-9^b^ | | | |
|  |  | B | SE | t | *P* value | B | SE | t | *P* value |
|  | |  |  |  |  |  |  |  |  |
| Baseline measure | | 0.84 | 0.003 | 246.40 | <.001 | 0.81 | 0.003 | 255.24 | <.001 |
| Timepoint | | –0.60 | 0.04 | –16.40 | <.001 | –0.60 | 0.04 | –15.52 | <.001 |
| Days on program | | 0.005 | 0.001 | 7.22 | <.001 | –0.004 | 0.001 | 5.39 | <.001 |
| Anxiety (=Yes) | | 0.57 | 0.05 | 12.44 | <.001 | 0.53 | 0.05 | 11.53 | <.001 |
| Depression (=Yes) | | 0.41 | 0.04 | 9.74 | <.001 | 0.39 | 0.05 | 8.67 | <.001 |
| Activities per week (BS)^c^ | | –0.15 | 0.01 | –14.72 | <.001 | –0.18 | 0.01 | –16.24 | <.001 |
| Activities per week (WS)^d^ | | –0.12 | 0.01 | –12.33 | <.001 | –0.13 | 0.01 | –13.19 | <.001 |
| **Interactions** | | | | | | | | | |
|  | Timepoint*Activities (BS) | –0.07 | 0.01 | –5.63 | <.001 | –0.04 | 0.01 | –3.13 | .002 |
|  | Timepoint*Activities (WS) | 0.10 | 0.01 | 6.72 | <.001 | 0.13 | 0.02 | 8.28 | <.001 |

^a^GAD-7: generalized anxiety disorder.

^b^PHQ-9: patient health questionnaire.

^c^BS: between-subject.

^d^WS: within-subject.
