# Supplementary material for: The Effect of a Digital Mental Health Program on Anxiety and Depression Symptoms: Retrospective Analysis of Clinical Severity
Source: JMIR Form Res. 2023 Oct 3;7:e36596. doi: 10.2196/36596 (PMC10582814; doi:10.2196/36596)
Supplement: Multimedia Appendix 2 [file formative_v7i1e36596_app2.docx]

Results of mixed effects models testing the effect of activity engagement on GAD-7^a^ and PHQ-9^b^ scores for participants with severe scores on full sample.

| Characteristic | | Measure (Outcome) | | | | | | | |
| --- | --- | --- | --- | --- | --- | --- | --- | --- | --- |
|  |  | GAD-7^a^ (N=3226) | | | | PHQ-9^b^ (N=1652) | | | |
|  |  | B | SE | t | *P* value | B | SE | t | *P* value |
|  | |  |  |  |  |  |  |  |  |
| Baseline measure | | 0.84 | 0.03 | 28.20 | <.001 | 0.80 | 0.04 | 19.73 | <.001 |
| Timepoint | | –2.61 | 0.10 | –25.14 | <.001 | –3.29 | 0.16 | –20.16 | <.001 |
| Days on program | | 0.02 | 0.002 | 9.27 | <.001 | 0.03 | 0.003 | 8.10 | <.001 |
| Anxiety (=Yes) | | 0.66 | 0.31 | 2.16 | .03 | 0.58 | 0.33 | 1.77 | .08 |
| Depression (=Yes) | | 0.64 | 0.16 | 4.03 | <.001 | 0.76 | 0.45 | 1.70 | .09 |
| Activities per week (BS)^c^ | | –0.31 | 0.03 | –10.64 | <.001 | –0.31 | 0.05 | –6.427 | <.001 |
| Activities per week (WS)^d^ | | –0.26 | 0.03 | –10.30 | <.001 | –0.32 | 0.04 | –7.73 | <.001 |
| **Interactions** | | | | | | | | | |
|  | Timepoint*Activities (BS) | –0.02 | 0.03 | –0.60 | .55 | 0.04 | 0.06 | 0.59 | .55 |
|  | Timepoint*Activities (WS) | 0.21 | 0.04 | 5.18 | <.001 | 0.17 | 0.08 | 2.21 | .03 |

^a^GAD-7: generalized anxiety disorder.

^b^PHQ-9: patient health questionnaire.

^c^BS: between-subject.

^d^WS: within-subject.
